# Three‐Dimensional Presentation of Tongue Squamous Cell Carcinoma Histopathology and Magnetic Resonance Imaging—A Novel Image Fusion Method

**DOI:** 10.1002/hed.70032

**Published:** 2025-09-04

**Authors:** Anne Koivuholma, Heli J. Sistonen, Katri Aro, Alexandria L. Irace, Antti Mäkitie, Jaana Hagström, Timo Atula

**Affiliations:** ^1^ Department of Otorhinolaryngology‐Head and Neck Surgery University of Helsinki and Helsinki University Hospital Helsinki Finland; ^2^ Department of Pathology, HUSLAB Helsinki University Hospital and University of Helsinki Helsinki Finland; ^3^ Department of Radiology University of Helsinki and Helsinki University Hospital Helsinki Finland; ^4^ Department of Otorhinolaryngology‐Head and Neck Surgery University of Pennsylvania Health System Philadelphia Pennsylvania USA; ^5^ Division of Ear, Nose and Throat Diseases, Department of Clinical Sciences, Intervention and Technology Karolinska Institutet and Karolinska University Hospital Stockholm Sweden; ^6^ Research Program in Systems Oncology, Faculty of Medicine University of Helsinki Helsinki Finland; ^7^ Department of Oral Pathology and Radiology‐Institute of Dentistry University of Turku Turku Finland

**Keywords:** cancer, computer aided design, histopathology, magnetic resonance imaging, oral squamous cell carcinoma, three‐dimensional reconstruction, tongue

## Abstract

**Background:**

Three‐dimensional (3D) modeling has been used in the management of bony head and neck tumors, but not in soft tissue tumors. Currently, histopathological findings of oral squamous cell carcinomas (OSCC) are presented as two‐dimensional images. Previously, we developed a 3D image fusion method that presents tumor histopathology and MRI in 3D form. In this study, we sought to test our method in a series of OSCCs.

**Methods:**

A 3D table scanner, 3D Slicer software, and 3D modeling software were used to produce 3D image fusions of nine OSCCs.

**Results:**

Nine OSCCs are presented as digital, 3D image fusions, showing the resected specimen and the tumor within based on histopathology and MRI.

**Conclusions:**

The fused 3D model allows for a better visual understanding of tumor orientation within the resected specimen, provides a tool to compare tumor volume and dimensions between MRI images and histopathology slides, and facilitates multidisciplinary discussion.

**Abbreviations**
3D: three‐dimensional
ADC: apparent diffusion coefficient
AI: artificial intelligence
OSCC: oral squamous cell carcinoma

## 1 Introduction

Three‐dimensional (3D) modeling is a valuable tool in the evaluation and management of bony tumors in head and neck surgery. For example, virtual 3D models and cutting guides are commonly used to assist with mandibular tumor resections and free flap reconstruction [1]. However, 3D modeling of soft tissue specimens has not been explored to the same degree. After resection, the orientation of the tumor and location of tumor histopathological margins are difficult to visualize due to deformation of the soft tissue. In current practice, only 2D images and histological slides of specimens are available postoperatively to represent the tumor's original orientation and topography. This makes it challenging to identify the exact location of tumor histopathological margins in the resected specimen, which in turn makes it difficult to precisely pinpoint areas of interest when considering additional surgery or radiation therapy. Furthermore, comparing histopathological findings to MRI is difficult because imaging slices are from a different plane and of different width than histopathological slices.

Previous research has demonstrated successful soft tissue 3D modeling using a printed mold, plate, or guide from a preoperative MRI [2, 3, 4, 5]. These studies have mainly focused on prostate specimens and have solely used CT or MRI to create the 3D model, without any histopathological component. Histopathological 3D models have also been constructed from histopathology images slice by slice and compared to preoperative MRI scans in prostate and breast cancer [6, 7]. Because these methods are robust and time‐consuming, as well as not directly applicable to other tissue types, such as head and neck tumor specimens, further development is needed. Furthermore, they do not allow the pathologist to freely determine dissection directions (i.e., the pathologist's planned cuts) of tumor specimens because they require the specimen to be sliced from end to end, whereas typically the pathologist prepares slides only from spots of interest.

As a proposed solution, 3D optical scanning is an emerging method of depicting resected tumor specimens. This approach has been used in constructing a 3D model of OSCC histopathology [8] and head and neck surgical specimen mapping [9, 10]. In addition, our previous work successfully developed a method using optical 3D scanning and 3D computer‐aided (CAD) modeling for presenting tongue tumor histopathology and MRI in a fused 3D model [11]. The purpose of this study was to examine the replicability of our method in a series of resected OSCC specimens (*n* = 9; Table 1). To the best of our knowledge, this type of “fused” 3D model has not been previously presented by other groups.

**TABLE 1:** Nine collected tongue squamous cell carcinoma patient data and tumor volume measurements.

	Gender/age^a^	Tumor	TNM classification	MRI volume (cm^3^)	Histopathological volume (cm^3^)	Difference (cm^3^)	Histopathological model vs. MRI model (%)^f^	Number of histopathological slides
1	M/68	Recurrent^b^	cT3N1M0/rpT3rpN2b	8.45	5.08	3.4	60.1	7
2	F/68	Primary	cT2N0M0/pT2pN0	1.60	0.60	1.0	37.5	8
3	M/54	Primary	cT3N2cM0/pT3pN0	8.20	4.50	3.7	54.9	8
4	F/57	Residual^c^	cT3N2cM0/pT4apN1	12.60	1.10	11.5	8.7	9
5	M/74	Second primary^d^	cT2N1M0/pT3pN1	0.60	0.40	0.2	66.7	12
6	M/46	Primary	cT2N0M0/pT1pN0	2.20	2.40	−0.2	109.1	12
7	F/52	Primary	cT2N3bM0/pT2pN3b	3.90	2.60	1.3	66.7	16
8	F/55	Primary	cT2N2bM0/pT3pN1	4.00	4.00	0.0	100.0	21
9	M/72	Second primary^e^	cT2N2bM0/pT3pN0	4.90	4.30	0.6	87.8	11
			Median	4.0	2.6	1.0	66.7	
			Average	5.2	2.8	2.4	65.7	

^a^ M, male; F, female; age in years.

^b^ The patient had been operated on for a T1 tongue carcinoma 2.5 years earlier.

^c^ The patient had initially received chemoradiation (crt), surgery for residual disease 3 months after completion of crt.

^d^ The patient had received chemoradiation to a T3 supraglottic carcinoma 1.5 years earlier.

^e^ The patient had received chemoradiation to a T2 hypopharynx carcinoma 12 years earlier.

^f^ Calculated by dividing the histopathological volume by the MRI volume.

## 2 Materials and Methods

### 2.1 Sample Selection

Patients were recruited to the study prior to surgery if they were diagnosed with OSCC of the tongue with a tumor diameter measuring ≥ 2 cm. All included patients underwent a preoperative MRI, which was performed according to the routine imaging protocol at our institution, with adequate visualization of the anatomic borders of the tumor and no significant artifacts. The study cohort consisted of nine patients whose data are presented in Table 1. Of note, one previous case was used to develop and demonstrate the method as described in Koivuholma et al. [11]. Of the nine patients included, five were male and four were female, with a mean age of 58 years (range, 44–71). Three patients had total glossectomies (Cases 1, 3, 4), and six had partial glossectomies (Cases 2, 5–9). One patient had a recurrent tumor, as a T1 tongue carcinoma had been surgically managed 2.5 years earlier (Case 1). One patient had a residual tumor after chemoradiotherapy (Case 4). One patient had a history of chemoradiated T3 supraglottic carcinoma (Case 5). Another patient had survived a T2 hypopharynx carcinoma 12 years earlier (Case 9). Suitable anatomic landmarks were selected individually for each case, which were later used to help orient and superimpose the radiologic and histopathological models to create a fused 3D model. The selected landmarks included the tongue midline in five cases (1, 2, 3, 4, and 6), sublingual gland in two (2 and 7), palatal arch in one (5), and palatine tonsil in two cases (8, 9).

### 2.2 Creating 3D Models of Tumor

Eight patients were imaged with 1.5 T Siemens Avanto dot (Siemens Healthcare, Erlangen, Germany) and one patient with 1.5 T Siemens MAGNETOM Sola (Siemens Healthcare, Erlangen, Germany). The diffusion images were obtained in an axial plane with a slice thickness of 4 mm using a readout‐segmented echo‐planar imaging sequence (RESOLVE). The RESOLVE technique segments the *k*‐space trajectory along the readout direction, enhancing susceptibility sensitivity, reducing image distortion, and improving image quality for better lesion detection and delineation. Diffusion weighting was applied in three orthogonal directions, using *b*‐values of 50, 400, and 1000 s/mm^2^, 50, 500, and 1000 s/mm^2^, or 50 and 1000 s/mm^2^, depending on the MRI system. The apparent diffusion coefficient (ADC) map was calculated using all *b*‐values. A single‐shot echo‐planar imaging technique with a slice thickness of 5 mm was used to acquire the diffusion imaging stack of the lower neck.

In addition to the diffusion images, the imaging protocol included axial T2‐weighted imaging (T2WI), axial fat‐saturated T2WI, axial T1‐weighted imaging (T1WI), axial fat‐saturated contrast‐enhanced T1WI, coronal T2WI, and coronal fat‐saturated contrast‐enhanced T1WI. Apart from diffusion images, the upper stack (including tumor area and upper neck axial images) was obtained with a slice thickness of 3 mm, and the lower stack with a slice thickness of 4 mm. The 3D tumor MRI models were created by segmenting the MRI stack using 3D Slicer. Tumor segmentation means that the radiologist determines tumor edges image by image for the creation of the 3D MRI tumor model. Tumor segmentation was performed manually by an experienced ENT radiologist (H.J.S.) in ADC maps including only the areas that indicated restricted diffusion, i.e., areas that were bright in diffusion trace images (*b*‐value 1000 s/mm^2^) and dark in ADC. We selected the diffusion imaging ADC map for tumor segmentation because it most accurately distinguishes malignant tissue from surrounding reactive changes, preventing overestimation of tumor size [12]. The fat‐saturated contrast‐enhanced T1WI and fat‐saturated T2WI aided in distinguishing tumor extent. Segmentation was done retrospectively; the 3D MRI model was not available preoperatively.

### 2.3 Creating 3D Models of Resection Specimen and Tumor Histopathology

The 3D tumor histopathological models were developed using our previously reported method [8]. The approach requires a table scanner (Einscan SP, Shining 3D), digital microscopy slides (Zen 3.0 Carl Zeiss Microscopy GmbH, Oberkochen, Baden‐Wurttemberg, Germany) and 3D modeling software (Fusion, v2021‐2023, Autodesk Inc. Mill valley, CA, USA). In this method, the specimen is scanned by a 3D table scanner immediately after resection. The specimen is placed on a net suspended from a simple rack to ensure that the surface geometry is captured from all angles. By suspending the net, a hammock‐like surface is created for the resection specimen to limit deformation. Given our sample included tumor specimens of different sizes, the rack was able to be adjusted to facilitate larger specimens.

After modeling, the specimen is then handled according to the normal protocol by the pathologist (J.H.) and histopathological slides are prepared. The number of slides varied case by case and was determined by the examining pathologist (J.H.) according to normal protocol. The microscopy slides were digitalized, and tumor outlines were drawn onto the digital slides by the pathologist (J.H.). The 3D tumor within the specimen was modeled using the tumor outlines and the scanned specimen in 3D modeling software (Fusion) by a member of the research team (A.K.). The histopathological 3D model is derived from the tumor outlines drawn by the pathologist. The pathologist gave final tumor margins based on histopathological H&E staining and microscopy. Frozen section analysis was done in cases 4, 5, 6, 7, and 8, and they were negative.

### 2.4 Creating the Image Fusions

**FIGURE 1:**
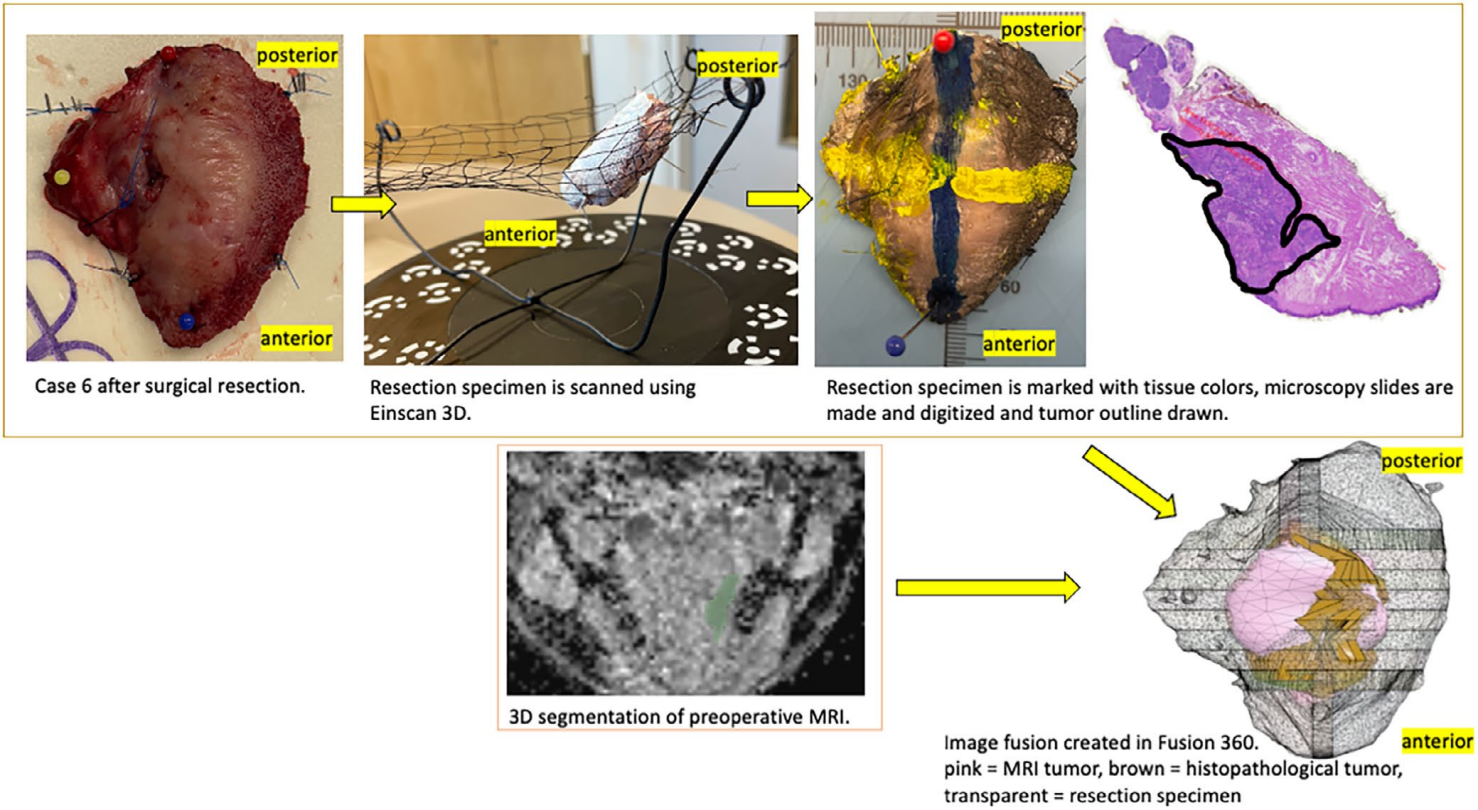
Workflow of creating a 3D image fusion of resection specimen, MRI, and histopathology tumors. [Color figure can be viewed at wileyonlinelibrary.com]

Suitable anatomical landmarks (described above) were marked on the specimen using pins in the operating room by the operating surgeon. The radiologist made corresponding markings on the 3D MRI model in 3D Slicer. The 3D MRI tumor models along with their coordinates and anatomical landmarks were exported from 3D Slicer in .stl form into Fusion. Respective anatomical landmarks were used to superimpose the 3D MRI and 3D histopathology tumor models as accurately as possible in Fusion. We created this method of presenting a 3D image fusion of tumor histopathology and MRI in our previous work [11]. The workflow is presented in Figure 1. Tumor dimensions in the anterior–posterior (A/P), cranial–caudal (C/C), and left–right (L/R) direction were measured using the histopathological as well as the radiological 3D models for each case.

## 3 Results

**FIGURE 2:**
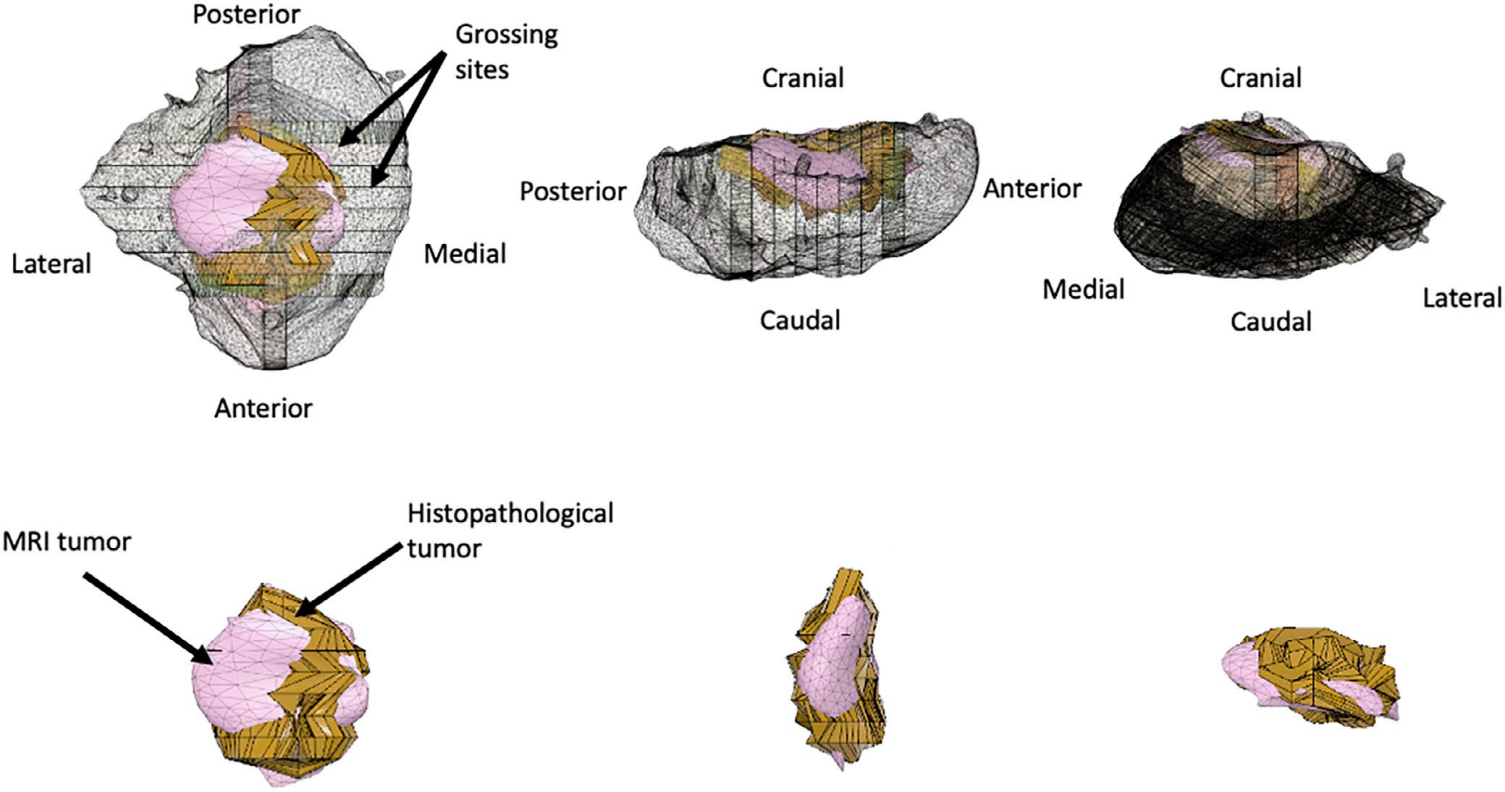
Demonstration of case 6 resection specimen, histopathological and MRI tumor. [Color figure can be viewed at wileyonlinelibrary.com]

As a result, all cases are presented as digital, 3D image fusions, showing the resected specimen and the tumor within. The tumor is presented as two bodies, one defined by the histopathology and the other by MRI. Case 6 demonstrates our method in detail and is presented in Figure 2, as well as in video format (Video S1, duration 1.5 min). All patients are demonstrated in video format (Video S2, duration 4 min).

Some of the cases had special characteristics that needed to be addressed in MRI segmentation. Tonsillar tissue and salivary glands typically have restricted diffusion that is very similar to malignant tumors. In three patients, palatine or lingual tonsil or sublingual salivary gland abutted the tumor and was somewhat difficult to differentiate from it (Cases 2, 7, 9). In these cases, other sequences were used to aid in segmentation. In case 4, the tumor was ulcerated, and the ulcerated pit was excluded from the MRI segmentation as it did not contain tissue. In case 5, the tumor was small and superficial at the posterior aspect of the tongue, which made segmentation more difficult.

Comparisons between histopathological and radiological model measurements in A/P, C/C, and L/R directions and 3D model volumes are presented in Table 2. The histopathological measurements were based on formalin‐fixed resection specimens. The estimated tumor volume was greater in the MRI model than the histopathological model in seven cases (Cases 1–5, 7, and 9), smaller in one case (Case 6) and equal in one case (Case 8). The MRI model yielded a higher A/P measurement in five cases (Cases 1, 2, 4, 6, and 7), lower in three cases (Cases 3, 8, and 9), and equal in one case (Case 5). The MRI model yielded higher C/C and L/R measurements in seven cases (Cases 2–7 and 9) and lower measurements in two cases (Cases 1 and 8). The largest tumor dimension in the MRI models was measured as the greatest distance between two points on the tumor surface. This measurement was a combination of different directions (A/P, C/C, and L/R). Because of this, the largest tumor dimension was greater than the other measurements in cases 2, 3, 4, 5, 7, and 8.

**TABLE 2:** Measurement details per case.

Patient	MRI model (cm)	Histopathological model (cm)	Difference (cm)	Percentual change (%)^a^
Tumor anterior/posterior dimensions				
1	3.7	1.9	1.8	49.6
2	2.1	2.6	−0.5	−24.1
3	4.0	4.2	−0.2	−4.2
4	3.9	0.7	3.2	82.1
5	2.0	2.0	0.0	0.0
6	2.8	2.7	0.1	3.2
7	3.2	2.7	0.5	14.5
8	3.1	3.2	−0.1	−2.9
9	2.8	3.5	−0.7	−26.0
Tumor cranial/caudal dimensions				
1	3.0	3.3	−0.3	−9.0
2	2.0	1.6	0.4	19.4
3	4.3	2.2	2.1	48.4
4	5.8	3.4	2.4	41.1
5	1.6	1.4	0.2	14.8
6	2.5	2.4	0.1	4.1
7	2.1	1.9	0.3	12.2
8	2.7	2.9	−0.2	−7.9
9	2.9	2.6	0.2	8.4
Tumor left/right dimensions				
1	3.4	3.7	−0.4	−11.2
2	0.9	0.7	0.2	24.2
3	2.2	3.9	−1.7	−76.6
4	2.8	2.1	0.7	25.0
5	0.4	0.3	0.1	13.9
6	1.3	1.3	0.0	0.8
7	1.6	1.1	0.6	34.0
8	2.0	1.5	0.6	27.4
9	1.8	1.6	0.2	13.1

Patient	Largest dimension on MRI (cm)	Largest dimension on microscopy slide (cm)	Difference (cm)	Percentual difference (%)
Largest tumor dimensions				
1	3.7	3.7	0.0	0.3
2	2.7	1.6	1.1	41.9
3	4.4	2.8	1.6	35.5
4	6.3	2.6	3.7	58.8
5	2.7	2.0	0.8	28.3
6	2.8	2.7	0.1	3.6
7	3.4	2.8	0.6	17.9
8	3.2	3.3	−0.1	−3.4
9	2.9	3.3	−0.5	−16.4

^a^ Calculated by subtracting the histopathological measurement from the respective MRI measurement and dividing the result by the MRI measurement.

## 4 Discussion

In this study, we applied our previously reported method [11] to produce a 3D fused model of tumor conformation based on histopathological analysis and preoperative MRI for nine tongue OSCC specimens. We found that our method is, at least moderately, applicable to partial and total glossectomy resection specimens. The method allows the pathologist to choose free grossing directions as in the standard protocol. Additional equipment required includes only a table scanner and 3D modeling software. Comparing tumor MRI to 2D histopathology is difficult because the microscopic tissue slices and MRI are not from corresponding planes. Our method may offer a more understandable method to present where exactly the tumor is situated in the tissue block and identify close margins in relation to the patient's anatomy and adjacent structures. This could be helpful when planning adjuvant therapies and the need for additional surgery, especially in cases where MRI findings are inconclusive. Image fusions including MRI and CT have been shown to improve interdisciplinary discussion [13, 14, 15]. Additionally, previous research has demonstrated that 3D scanning can be used intraoperatively to improve frozen section analysis and communication between the operating head and neck surgeon and pathologist [16, 17, 18]. The next step would be to incorporate the 3D image fusion results into the multidisciplinary tumor board meeting and investigate its usefulness further.

Our series demonstrates that the 3D MRI model typically yielded greater tumor volume and dimensional measurements (A/P, C/C, and L/R) compared with the 3D histopathological model. This is consistent with existing research. For instance, Lam et al. [19] have shown that when comparing oral SCC tumor dimensions between MRI and histopathology, MRI produces slightly larger dimensions. Similar findings have also been reported by other groups [20, 21].

One potential cause of this discrepancy is the accuracy of MRI segmentation, which may be affected by imaging artifacts, physiologic or post‐therapy changes like inflammation, or simply by human interpretation error [22]. This subsequently leads to a greater estimation of 3D tumor volume. To address this challenge, we segmented the tumors on the ADC map, which allows for more reliable differentiation of the tumor from surrounding inflammation and edema. In our series, one patient (Case 4) demonstrated a large difference between histopathological and MRI tumor measurements. In this case, the investigating pathologist (J.H.) reported abundant inflammation present on microscopy slides despite a much smaller tumor size. Further, this patient had undergone chemoradiation 3 months prior to resection, and the residual tumor was ulcerated. While there are no previous studies comparing the volumes of 3D models created based on diffusion images and histopathology, diffusion‐weighted imaging has been demonstrated to effectively differentiate residual tumor from benign post‐radiotherapy changes in the head and neck [23, 24, 25, 26]. In other anatomic locations, however, some types of dense fibrosis and inflammation due to radiation may result in restricted diffusion and therefore obscure true tumor extent [27]. MRI in case 4 showed prominent ulceration that was bordered by a zone of restricted diffusion, which was more expansile at the deepest part of the ulceration. ADC was uniformly low, 0.9–1.0 × 10^−3^ mm^2^/s, indicative of residual tumor, and the area was segmented in whole. In the same spot, a low T2 signal suggested post‐radiotherapy fibrosis, while abnormal enhancement across most of the tongue was consistent with inflammation. In retrospect, only the expansile part might have represented the residual tumor, with the other area representing post‐treatment changes. This case illustrates how MRI segmentation following radiotherapy can overestimate tumor volume, highlighting its reduced reliability in such scenarios. The effect of inflammation and fibrosis may be better demonstrated in a fused model using MRI and histopathology sectioning compared to preoperative imaging or a pathology report in isolation. In addition, human error may be mitigated by limiting MRI interpretation to one radiologist with expertise in head and neck anatomy. In this study, one experienced ENT radiologist (H.J.S.) performed all tumor segmentations. Multiple radiologists determining tumor extent may introduce variability based on their level of expertise and familiarity with relevant anatomy [28, 29, 30].

Another possible contributor to the discrepancy in tumor dimensions between the two models is tissue shrinkage and deformation during postoperative handling [21, 22, 28, 29, 31, 32]. Although the tumor specimen is fixed in formalin prior to slicing and histological analysis, which should theoretically preserve protein architecture prior to manipulation by the pathologist, it is possible that the handling of the tissue alters the gross shape or size of the tumor. Additionally, it has been shown that formalin fixation may result in 4%–6% of tissue shrinkage [33]. These factors may alter the 3D histopathological measurements and lead to decreased estimated dimensions. We used a simple rack to limit the tissue deformation during scanning. This rack was able to accommodate both total and partial glossectomy specimens. Still, the specimen is not in the same position during scanning as it is in the human body. Further work is needed to tackle this problem. One solution could be to use point cloud registration and digital twins to estimate tissue deformation and adjust the scanned 3D body accordingly. This is a new, emerging artificial intelligence (AI) ‐based method that can help measure tissue deformation presented by Monji‐Azad et al. [34]. In future studies, the accuracy of MRI segmentation could be enhanced by selecting patients without prior radiotherapy, and by improving image resolution through increased slice thickness or the use of 3D sequences.

The construction method of the 3D histopathological model may also affect the tumor volume. The 3D model is based on tumor outlines of adjacent microscopy slides. The tumor volume is interpolated from these outlines; thus, the 3D histopathological tumor model is an approximation of the tumor volume.

In the future, AI could be used to automate our proposed method. For instance, AI may be able to construct the histopathological model from digitalized microscopy slides as well as segment MRI. This minimizes the need for human interpretation, thereby reducing human error, improving accuracy, and expediting processing. Deep learning has been successfully used in the segmentation of nasopharyngeal carcinoma [35]. This technology could potentially be applied to create 3D models based on preoperative imaging and postoperative histopathology slides of various tumors.

## 5 Conclusions

We used nine tongue OSCC resection specimens to evaluate the replicability of developing fused 3D tumor models based on preoperative imaging and histopathological analysis. We successfully created 3D image fusions to show that our method is suitable for partial and total glossectomy resection specimens. This fused model may yield improved visualization of tumor location and orientation within the resected specimen, provide a tool to compare tumor size and topography between MRI images and histopathology slides, and facilitate multidisciplinary postoperative discussion regarding adjuvant therapies and additional surgery. Further research should focus on the application of AI to help standardize and expedite the development of 3D soft tissue tumor models, not limited only to head and neck tumors.

## Ethics Statement

The Research Ethics Board of the Hospital District of Helsinki and Uusimaa approved the study protocol (record number: HUS/15/2024 approval date 7.2.2024, HUS/1092/2018 approval date 9.5.2018), and institutional permit was granted. An informed, written consent was obtained from recruited participants. We confirm that all experiments were performed in accordance with the Declaration of Helsinki.

## Consent

An informed, written consent was obtained from the participants to use images.

## Conflicts of Interest

The authors declare no conflicts of interest.

## Supporting information


**Video S1:** Demonstration of the 3D image fusion method of Case 6.

**Video S2:** 3D image fusions of nine tongue squamous cell carcinomas.

## Data Availability

The data that support the findings of this study are available from the corresponding author upon reasonable request.
